# Effect of maternal growth monitoring knowledge on stunting, wasting and underweight among children 0–18 months in Tamale metropolis of Ghana

**DOI:** 10.1186/s13104-020-4910-z

**Published:** 2020-01-29

**Authors:** Mohammed Bukari, Muzamil Mohammed Abubakari, Mohammed Majeed, Abdul-Razak Abizari, Anthony Wemakor, Ambrose Atosona

**Affiliations:** Department of Nutritional Sciences, School of Allied Health Sciences, University for Development Studies, P. O. Box TL 1883, Tamale, Ghana

**Keywords:** Growth monitoring, Knowledge, Wasting, Stunting, Underweight, Tamale

## Abstract

**Objective:**

This study sought to assess maternal growth monitoring knowledge and its effect on stunting, wasting and underweight among children 0–18 months in the Tamale Metropolis. An analytical cross-sectional study design, involving 340 mother–child pairs randomly selected from 4 health facilities in the Tamale Metropolis was used. A structured questionnaire was used to collect information on socio-demographic characteristics and maternal growth monitoring knowledge. Weight and length of children were taken to assess nutritional status (stunting, underweight and wasting). Chi square/Fisher’s exact test was used to determine the association between maternal growth monitoring knowledge level and child nutritional status.

**Results:**

The study revealed that 87.6% of mothers had good knowledge on growth monitoring. The prevalence of stunting, underweight and wasting were 9.4%, 25.9% and 17.9% respectively. Bivariate analysis revealed that there is no association between maternal growth monitoring knowledge and stunting (p = 0.781), wasting (p = 0.743) and underweight (p = 0.529) among children 0–18 months in the Tamale Metropolis.

## Introduction

Child growth monitoring is aimed at improving and maintaining child health and nutrition [1]. It is so far the best strategy carried out worldwide to tackle malnutrition [2]. It involves regular measurement and plotting of a child’s weight on a growth chart [3, 4]. Growth monitoring is a process of regularly following the growth rate of a child in comparison to standard anthropometric measurements [2]. It is usually coupled with promotional activities such as counselling and actions to improve growth, as weighing and charting alone cannot improve growth [3, 4]. Assessing growth allows capturing growth faltering before the child reaches the status of under-nutrition thereby reducing the risk of death [5]. According to Schwinger et al. [6], assessing a child’s growth trajectory provides a more accurate prognosis of likely death than the use of static measures of nutritional status. Sinaga et al. [7] also revealed that growth monitoring has a positive impact on infant feeding practices and calorie intake.

Growth monitoring is practiced worldwide [8] and its coverage in Ghana is 95.3% [9]. A79% coverage has also been reported in the Tamale Metropolis [10]. Despite its wide coverage, the prevalence of malnutrition is still high, indicating that it may be poorly implemented. Globally, approximately half of all deaths in under five children are link to malnutrition, translating into the loss of about 3 million lives a year [5].The prevalence of stunting, wasting and underweight for children under 5 years are 19%, 5% and 11% respectively in Ghana and 33.1%, 6.3% and 20% respectively in Northern region [11]. Malnutrition puts children at greater risk of dying from common infections, increases the frequency and severity of such infections, and delays recovery [5].

It has been established in other settings that poor knowledge on growth monitoring among mothers is associated with poor implementation of growth monitoring [9, 12] which results in high malnutrition rate. However, growth monitoring knowledge among mothers and its relation to child nutritional status is unknown in the Tamale Metropolis. Hence this study sought to assess maternal growth monitoring knowledge and its effect on nutritional status of children 0–18 months in the Tamale Metropolis.

## Main text

### Methodology

#### Study design, study setting and sampling

An analytical cross-sectional study design was employed and involved 340 mother–child pairs selected from 4 health facilities (Tamale Central Hospital, Reproductive and Child Health Centre, Nyohini Health Centre and Bulpiela Health Centre) in the Tamale Metropolis. The study was carried out from May 10 to June 15, 2018. Simple random sampling method was used in selecting the 4 study health facilities from all (10) the public health facilities in the Tamale Metropolis. Mothers with children aged 0–18 months attending the child welfare clinics of the selected health facilities were eligible for the study. Subjects who were very ill (unstable vital signs/mental status) were excluded from the study.

Sample size was determined using the formula [13]:

$${\text{N}}\, = \,\frac{{z^{2} p\,\left( {1 - p} \right)}}{{M.E^{2} }}$$

where N is the sample size; z is the abscissa of the normal curve that cut-off an area at the tail (1.96); P is the estimated proportion of an attribute (stunting) that is present in the study population, which is 33.1% [11] in Northern region; M.E is the desired level of precision (5% = 0.05).

$${\text{N}}\, = \,\frac{{\left( {1.96} \right)^{2} 0.331\left( {1 - 0.331} \right)}}{{\left( {0.05} \right)^{2} }}\, = \,340$$

Therefore, sample size for the study was 340 respondents.

#### Data collection

A pretested structured questionnaire was used to document information on participants’ demographic characteristics (sex of child, age of mother and child, mothers’ educational level, religion, occupation and marital status) and level of maternal knowledge on growth monitoring. Regarding growth monitoring knowledge, the questionnaire items were adapted from similar studies (9, 14). A composite score was on mothers’ responses to the questions. A score of 1 (maximum score) was assigned to a correct response while a score of 0 (minimum score) was assigned to an incorrect response. Mothers who scored 0–4 for the knowledge questions were considered to have poor knowledge while those who scored 5–8 for the knowledge questions were considered to have good knowledge. Anthropometric measurements (length and weight) of the children were taken and nutritional status (stunting, wasting and underweight) assessed in accordance with WHO standards [14]. Length of children was measured using an infantometre to the nearest 0.1 cm. Weight of children was measured using a digital electronic weighing scale (Seca 874) to the nearest 0.1 kg. Height-for-age Z-score (HAZ), weight-for-height Z-score (WHZ), and weight-for-age Z-score (WAZ) < − 2 standard deviations (− 2SD) from the median of the WHO Growth Standards were defined as stunting, wasting, and underweight respectively.

#### Statistical analysis

Data entry and analysis was done using Statistical Package for Social Sciences (SPSS) (IBM, version 21) and WHO Anthro software. Chi square/Fisher’s exact test was used to determine the association between maternal growth monitoring knowledge level and child nutritional status (stunting, wasting and underweight). p < 0.05 was considered significant at two tailed tests. Percentages and cross tabulations were used to show respondents’ responses.

### Results

#### Socio–demographic characteristics of the study sample

The mean age of mothers was 29.26 ± 5.64 years with the minimum and maximum ages of 17 and 50 years respectively. Most of the mothers (44.7%) had no formal education, with 96.8% being married. Moreover, majority of the mothers were self-employed (71.8%), belonged to Dagomba ethnic group (80.9%) and Islamic religion (93.8%). The mean age of children was 5.7 ± 4.3 months, with majority of them in the age group 0–5 months (57.4%). Also, majority of the children were males (51.5%). Table 1 depicts the socio-demographic characteristics of respondents.

**Table 1:** Socio-demographic characteristics

Characteristic	Category	Frequency (%)
Age of mother (years)	17–27	146 (42.9)
	28–37	158 (46.5)
	> 38	36 (10.6)
Education	None	152 (44.7)
	Primary	31 (9.1)
	Middle/JHS	59 (17.4)
	SHS/vocational training	51 (15)
	Tertiary	47 (13.8)
Occupation	Employed	38 (11.2)
	Self employed	244 (71.8)
	Unemployed	58 (17)
Ethnicity	Dagomba	275 (80.9)
	Gonja	19 (5.6)
	Mamprusi	11 (3.2)
	Others	35 (10.3)
Religion	Christianity	21 (6.2)
	Islam	319 (93.8)
Age of child (months)	0–5	195 (57.4)
	6–11	102 (30)
	12–18	43 (12.6)
Sex of child	Male	175 (51.5)
	Female	165 (48.5)

#### Maternal growth monitoring knowledge level

Table 2 shows maternal growth monitoring knowledge level. Majority of the mothers referred to weighing (80.6%) as the meaning of growth monitoring. A large section (85.6%) of the mothers said growth monitoring is important as it determines the child’s nutritional status. Also, majority (92.9%) said every mother ought to attend growth monitoring sessions once every month. Some of the mothers (31.8%) said the growth chart represents the growth of the child. With regards to overall knowledge level of mothers, 12.4% had poor knowledge while 87.6% had good knowledge.

**Table 2:** Maternal growth monitoring knowledge

Characteristic	Category	Frequency (%)
When you hear growth monitoring, what comes to mind?	Weighing	274 (80.6)
	Immunization	62 (18.2)
	Treatment of malnourished children	4 (1.2)
Importance of growth monitoring	Determines child’s nutritional status	291 (85.6)
	Educates about appropriate child feeding	44 (12.9)
	Don’t know	4 (1.2)
	Not important	1 (0.3)
Number of times one is supposed to go for growth monitoring sessions	Once a month	316 (92.9)
	Twice every month	15 (4.4)
	Once every 6 months	4 (1.2)
	Don’t know	5 (1.5)
When is one to start going forgrowth monitoring sessions?	At birth	282 (82.9)
	At 1 month of age	53 (15.6)
	At 1 year of age	5 (1.5)
Age range for growth monitoring	Children < 2 years of age	208 (61.2)
	Children 0–5 years of age	110 (32.3)
	Don’t know	22 (6.5)
One responsible for growthmonitoring provision	Nurses	321 (94.4)
	Midwives	10 (2.9)
	Doctors	8 (2.4)
	Teachers	1 (0.3)
Where growth monitoring services are provided?	Hospitals	79 (23.2)
	Health centers	248 (72.9)
	Don’t know	13 (3.8)
What does a growth chart represent?	It shows the age of child	63 (18.5)
	It shows how the child is growing	107 (31.5)
	It has no meaning	28 (8.2)
	Don’t know	142 (41.8)
Knowledge level	Poor knowledge	42 (12.4)
	Good knowledge	298 (87.6)

#### Nutritional status of children

The mean HAZ, WHZ and WAZ were 0.1 ± 1.7, − 1.08 ± 1.75 and − 0.78 ± 1.4 respectively (Additional file 1). The prevalence of stunting, wasting and underweight were 9.4%, 25.9% and 17.9% respectively (Additional file 2). It was also revealed in the present study that stunting and underweight were significantly associated with maternal education (p = 0.008) and age of child (p = 0.003) respectively (Additional file 3).

#### Relationship between maternal growth monitoring knowledge and child nutritional status

It was revealed in the present study that the association between maternal growth monitoring knowledge and stunting (p = 0.781), underweight (p = 0.529) and wasting (p = 0.743) is statistically insignificant as shown in Table 3.

**Table 3:** Relationship between maternal growth monitoring knowledge and stunting, wasting and underweight

Characteristic	Stunting			
	Stunted N = 32 (%)	Not stunted N = 308 (%)	Total N = 340 (%)	p-value
Knowledge level				
Poor knowledge	3 (7.1)	39 (92.9)	42 (100)	0.781
Good knowledge	29 (9.7)	269 (90.3)	298 (100)	

Characteristic	Underweight			
	Underweight N = 61 (%)	Not underweight N = 279 (%)	Total N = 340 (%)	p-value
Knowledge level				
Poor knowledge	9 (21.4)	33 (78.6)	42 (100)	0.529
Good knowledge	52 (17.4)	246 (82.6)	298 (100)	

Characteristic	Wasting			
	Wasted N = 88 (%)	Not wasted 252 (%)	Total N = 340 (%)	p-value
Knowledge level				
Poor knowledge	10 (23.8)	32 (76.2)	42 (100)	0.743
Good knowledge	78 (26.2)	220 (73.8)	298 (100)	

### Discussion

With regards to knowledge level of mothers on growth monitoring, the study revealed that majority (80.6%) of respondents chose weighing as the meaning of growth monitoring. This finding is comparable to that of Debuo et al. [9] in Lawra District, Upper West Region of Ghana, where 70.3% of mothers chose weighing as the meaning of growth monitoring. This indicates that there has been a lot of education on growth monitoring among mothers in both districts. Apart from massive education, it could also mean that the health personnel used similar approaches in delivering information to mothers. The present study also revealed that 85.6% of mothers thought growth monitoring improves children’s nutritional status and subsequently growth. This finding is similar to findings of other studies [9, 15]. In addition, 92.9% of participants in the current study said that growth monitoring is provided every month. A similar study by Daniel et al. [15] in Ethiopia revealed that 32.1% of the respondents said growth monitoring services is provided every month. The difference in findings could be due to the fact that mothers in Ethiopia did not receive adequate education on interval of growth monitoring visits as compared to mothers in Ghana. It could also be due to differences in health policy implementation between Ghana and Ethiopia. Furthermore, 98.5% of the respondents know when growth monitoring services should begin. In a similar study [15], a higher percentage (68.2%) of mothers knew when growth monitoring services ought to begin. If mothers know when growth monitoring is supposed to begin then there is a high probability that mothers will start growth monitoring early and could reap the full benefits of growth monitoring services. This study also uncovered that 32.1% of mothers felt growth monitoring was for children between the ages of 0–5 years. This finding is also in consonance with that of Daniel et al. [15].

Regarding the overall knowledge level of mothers on growth monitoring, 87.6% of the mothers had good knowledge in the present study. Similarly, a good knowledge level was also reported in studies conducted in Ghana [9, 16] and other parts of the world [17, 18]. Contrarily, a study by Daniel et al. [15] revealed that 53% of mothers had poor knowledge on growth monitoring, which could be due to inadequate education on growth monitoring in this setting.

The prevalence of stunting in the sampled population was 9.4%. This result is much lower than the Northern regional prevalence of 33.1% reported in the most recent Ghana Demographic Health Survey (GDHS) in 2014 [11]. It is also lower than the national stunting prevalence of 18.7% reported by WHO in 2014 [19]. The present study was conducted among children aged 0–18 months while that of GDHS and WHO were among children under 5 years, this methodological difference could partly account for the lower prevalence recorded in the present study. It is also worth noting that the prevalence rates reported by GDHS and WHO were average figures for Northern region and Ghana respectively, suggesting that other places in the Northern region or Ghana could have lower prevalence rates, hence the finding of the present study. Moreover, the decreasing trend in stunting prevalence in the Northern region since 2011 could also account for the low prevalence reported in the present study. The prevalence of stunting decreased from 37.4% in 2011 [20] to 33.1% in 2014 [11]. Although the prevalence of stunting is on the decrease, the finding of the present study shows that it is still a public health concern in Northern region of Ghana. The prevalence rates of underweight and wasting were 25.9% and 17.9% respectively in the present study. Regarding underweight, the prevalence rate in the current study was higher than the prevalence rates in Northern region of Ghana (20%) [11] and Ghana as a whole (11.2%) [19]. Similarly, the prevalence of wasting in the present study was higher than the prevalence rates in Northern region (6.3%) [11] and Ghana (5%) [19]. Again, the difference in methodologies employed may partly explain the difference in findings between the present and previous studies. Also, the high prevalence rates of underweight and wasting in the present study could be an indication that enough emphasis has not been laid on interventions geared towards tackling underweight and wasting in the Metropolis. Although underweight among children under five is not considered public health concern in Ghana [20] and Northern region [11], this study has shown that underweight should be considered a public health concern for children between 0–18 months in the Tamale Metropolis.

Moreover, no association was established between maternal growth monitoring knowledge level and stunting, underweight and wasting. On the other hand, a study revealed a significant association between underweight (p = 0.02) and maternal growth monitoring knowledge [21]. The variation in findings could be attributed to differences in attitudes and practices of mothers in relation to growth monitoring in both settings. The finding of the present study shows that good maternal knowledge of growth monitoring does not automatically translates to improved child nutritional status, unless it is coupled with good attitude and practices of growth monitoring.

### Conclusion

Growth monitoring knowledge level of the mothers is high, but has no effect on stunting, wasting and underweight among children 0–18 months in the Tamale Metropolis.

### Limitations

The cross-sectional nature of the study does not provide a good basis for determining causality as both exposure and outcome were assessed simultaneously. Moreover, nutritional status of the mothers was not assessed. This notwithstanding, we trust the study shed some light on the effect of maternal growth monitoring knowledge on child nutritional status in the Tamale Metropolis.

## Supplementary information

**Additional file 1.** Nutritional status indicators.

**Additional file 2.** Nutritional status of children.

**Additional file 3.** Nutritional status by socio-demographic characteristics of respondents.

## Data Availability

The datasets used and/or analyzed during the current study are available from the corresponding author on reasonable request.

## Abbreviations

GDHS: Ghana Demographic Health Survey
HAZ: Height-for Age-Z-score
M. E: marginal error
WAZ: Weight-for-Age-Z-score
WHO: World Health Organization
WHZ: Weight-for-Height-Z-score
