# Supplementary material for: Effect of maternal growth monitoring knowledge on stunting, wasting and underweight among children 0–18 months in Tamale metropolis of Ghana
Source: BMC Res Notes. 2020 Jan 29;13:45. doi: 10.1186/s13104-020-4910-z (PMC6988331; doi:10.1186/s13104-020-4910-z)
Supplement: Supplementary file 1 — Additional file 1. Nutritional status indicators. [file 13104_2020_4910_MOESM1_ESM.docx]

**Additional file 1: Nutritional status indicators**

| **Indicator** | **Mean ± standard deviation** |
| --- | --- |
| Height-for age-z-score | 0.10±1.7 |
| Weight-for-height-z-score | -1.08±1.75 |
| Weight-for-age-z-score | -0.76±1.4 |
