# Supplementary material for: Effect of maternal growth monitoring knowledge on stunting, wasting and underweight among children 0–18 months in Tamale metropolis of Ghana
Source: BMC Res Notes. 2020 Jan 29;13:45. doi: 10.1186/s13104-020-4910-z (PMC6988331; doi:10.1186/s13104-020-4910-z)
Supplement: Supplementary file 2 — Additional file 2. Nutritional status of children. [file 13104_2020_4910_MOESM2_ESM.docx]

**Additional file 2: Nutritional status of children**

| **Characteristic** | **Category** | **Frequency (%)** |
| --- | --- | --- |
| Stunting | Stunted | 32 (9.4) |
|  | Normal | 308 (90.6) |
|  | Total | 340 (100.0) |
| Wasting | Wasted | 88 (25.9) |
|  | Normal | 252 (74.1) |
|  | Total | 340 (100.0) |
| Underweight | Underweight | 61 (17.9) |
|  | Normal | 279 (82.1) |
|  | Total | 340 (100.0) |
