# Supplementary material for: Effect of maternal growth monitoring knowledge on stunting, wasting and underweight among children 0–18 months in Tamale metropolis of Ghana
Source: BMC Res Notes. 2020 Jan 29;13:45. doi: 10.1186/s13104-020-4910-z (PMC6988331; doi:10.1186/s13104-020-4910-z)
Supplement: Supplementary file 3 — Additional file 3. Nutritional status by socio-demographic characteristics of respondents. [file 13104_2020_4910_MOESM3_ESM.docx]

**Additional file 3: Nutritional status by socio-demographic characteristics of respondents**

| **Characteristic** | **Stunting** | | **Wasting** | | **Underweight** | |
| --- | --- | --- | --- | --- | --- | --- |
|  | **Stunted n=32 (%)** | **P-value** | **Wasted n=88 (%)** | **P-value** | **Underweight**  **N=61 (%)** | **P-value** |
| **Age of mother** |  |  |  |  |  |  |
| 17-27 | 9 (28.1) | 0.193 | 39 (44.3) | 0.934 | 27 (44.3) | 0.688 |
| 28-37 | 19 (59.4) |  | 40 (44.5) |  | 26 (42.6) |  |
| >37 | 4 (11.1) |  | 9 (10.2) |  | 8 (13.1) |  |
| **Education** |  |  |  |  |  |  |
| None | 23(71.9) | 0.008 | 40(45.5) | 0.445 | 32(52.5) | 0.195 |
| Primary | 1(3.3) |  | 6(19.4) |  | 6(9.8) |  |
| Middle/JHS | 5(15.6) |  | 14(15.9) |  | 9(14.8) |  |
| SHS/vocational | 3(9.4) |  | 18(20.5) |  | 11(18) |  |
| Tertiary | 0(0.0) |  | 10(11.4) |  | 3(4.9) |  |
| **Occupation** |  |  |  |  |  |  |
| Self employed | 28(87.5) | 0.106 | 69(78.4) | 0.211 | 49 (80.3) | 0.240 |
| Employed | 1(3.1) |  | 6(6.8) |  | 4(6.6) |  |
| Unemployed | 3(9.4) |  | 13(17.9) |  | 8(13.1) |  |
| **Marital status** |  |  |  |  |  |  |
| Single | 1 (3.9) | 0.669 | 2 (2.3) | 0.612 | 2 (3.3) | 0.801 |
| Married | 31 (96.9) |  | 86 (97.7) |  | 59 (96.7) |  |
| **Age of child** |  |  |  |  |  |  |
| 0-5 | 15 (46.9) | 0.452 | 45 (51.1) | 0.158 | 24 (39.3) | 0.003 |
| 6-11 | 12 (37.5) |  | 27 (30.7) |  | 23 (37.7) |  |
| 12-18 | 5 (15.6) |  | 16 (18.2) |  | 14 (23) |  |
| **Sex of child** |  |  |  |  |  |  |
| Male | 20 (62.5) | 0.190 | 46 (52.3) | 0.861 | 33 (54.1) | 0.650 |
| Female | 12 (37.5) |  | 42 (47.7) |  | 28 (45.9) |  |
